# Ménétrier’s disease presenting as recurrent unprovoked venous thrombosis: a case report

**DOI:** 10.1186/s13256-018-1952-0

**Published:** 2019-01-17

**Authors:** H. Karl Greenblatt, Brave K. Nguyen

**Affiliations:** University of Cincinnati College of Medicine, Cincinnati, OH 45219 USA

**Keywords:** Thrombophilia, Hypercoagulable, Ménétrier’s, Gastropathy, Hypoalbuminemia

## Abstract

**Background:**

Acquired thrombophilia is a potential sequela of malignancy, chronic inflammation, and conditions characterized by severe protein deficiency (for example, nephrotic syndrome, protein-losing enteropathy). As such, venous thrombosis is often a feature, and occasionally a presenting sign, of systemic disease. Ménétrier’s disease is a rare hyperplastic gastropathy that may lead to gastrointestinal protein loss and hypoalbuminemia. To date, reports of venous thrombosis associated with Ménétrier’s disease are exceedingly scarce.

**Case presentation:**

We report the case of a 40-year-old white man who presented with unprovoked deep venous thrombosis, pulmonary embolism, and renal vein thrombosis. Upon receiving therapeutic anticoagulation, he developed severe gastrointestinal bleeding, and endoscopic evaluation led to a diagnosis of Ménétrier’s disease. A laboratory workup revealed deficiency of protein C, protein S, and antithrombin III, as well as markedly elevated levels of factor VIII. He was determined to have an acquired thrombophilia as a direct result of Ménétrier’s disease.

**Conclusions:**

This case describes an acquired thrombophilic state in a patient with Ménétrier’s disease and profound hypoalbuminemia. Although this association is rarely described, we discuss the probable mechanisms leading to our patient’s thrombosis. Specifically, we posit that his gastrointestinal protein loss led to a deficiency of several anticoagulant proteins and a compensatory elevation in factor VIII, as occurs in nephrotic syndrome and inflammatory bowel disease. Of note, this patient’s recurrent venous thrombosis was the initial clinical sign of his gastrointestinal pathology.

## Background

Thrombophilia refers to any condition that increases the risk of venous and/or arterial thrombosis, most commonly deep venous thrombosis (DVT) and pulmonary embolism (PE). Etiologies of thrombophilia may broadly be grouped into inherited conditions, including factor V Leiden and prothrombin gene mutation, and acquired conditions such as pregnancy, autoimmune disease, or malignancy. In patients with unprovoked DVT, especially those under age 50, laboratory evaluation for possible thrombophilia is usually not recommended, as it is unlikely to change medical management [1]. However, unprovoked DVT is occasionally the first presentation of an underlying illness. For example, Trousseau syndrome refers to a well-described association between unprovoked DVT and gastric or pancreatic carcinoma [2]. Therefore, in some patients with recurrent thrombosis or thrombosis in an unusual location (for example, renal vein or portal vein), further workup for the source of hypercoagulability may be indicated.

Ménétrier’s disease (MND) is a type of hyperplastic (or hypertrophic) gastropathy, first described by the French pathologist Pierre Ménétrier in 1888. To date, fewer than 1000 total cases have been reported. Its pathogenesis is driven by aberrant epidermal growth factor receptor (EGFR)-mediated signaling and overproduction of transforming growth factor alpha (TGFα) in the gastric mucosa. MND may be idiopathic but is strongly associated with *Helicobacter pylori* infection [3–5] and, in children, acute cytomegalovirus infection [6]. Typical presenting symptoms of MND are postprandial epigastric pain, early satiety, nausea/vomiting, and weight loss. The most frequently observed laboratory findings are hypoalbuminemia, hypochlorhydria, elevated serum gastrin, and iron deficiency anemia [7–12]. Imaging often reveals giant gastric folds, which have been said to resemble brain gyri [13]. Definitive diagnosis is made by biopsy; the most specific finding is foveolar hyperplasia without atypia or malignancy [14]. First-line treatment usually consists of a high-protein diet. Beyond this, the only satisfactory therapy has historically been total gastrectomy. More recently, several authors have reported regression of disease upon treatment with cetuximab, a monoclonal antibody against the EGFR receptor [15, 16]. MND is believed to be a premalignant condition, although the precise risk of progression to gastric carcinoma remains uncertain [17].

Although gastric carcinoma itself has been associated with thrombophilia, published reports of unprovoked thrombosis associated with MND or other hyperplastic gastropathies are exceedingly scarce [18–20]. This report presents the case of a patient who presented with DVT, PE, and renal vein thrombosis as the initial signs of MND. It will also discuss the mechanisms by which a protein-losing gastropathy such as MND could lead to a thrombophilic state.

## Case presentation

A 40-year-old white man presented to an outside emergency department in June 2018 with sudden-onset right calf pain, swelling, and redness. He had a history of juvenile polyposis syndrome, for which he underwent a partial colectomy as a child, and alcohol use disorder (in remission). He received surveillance colonoscopies at recommended intervals due to his history of polyps, the most recent of which had been unremarkable. Upon presentation, a venous ultrasound revealed an acute, occluding thrombus of his right popliteal, tibial, and peroneal veins. There was no preceding history of trauma or immobilization. He was discharged on rivaroxaban 15 mg twice daily and advised to follow-up with his primary care provider.

**Fig. 1:**
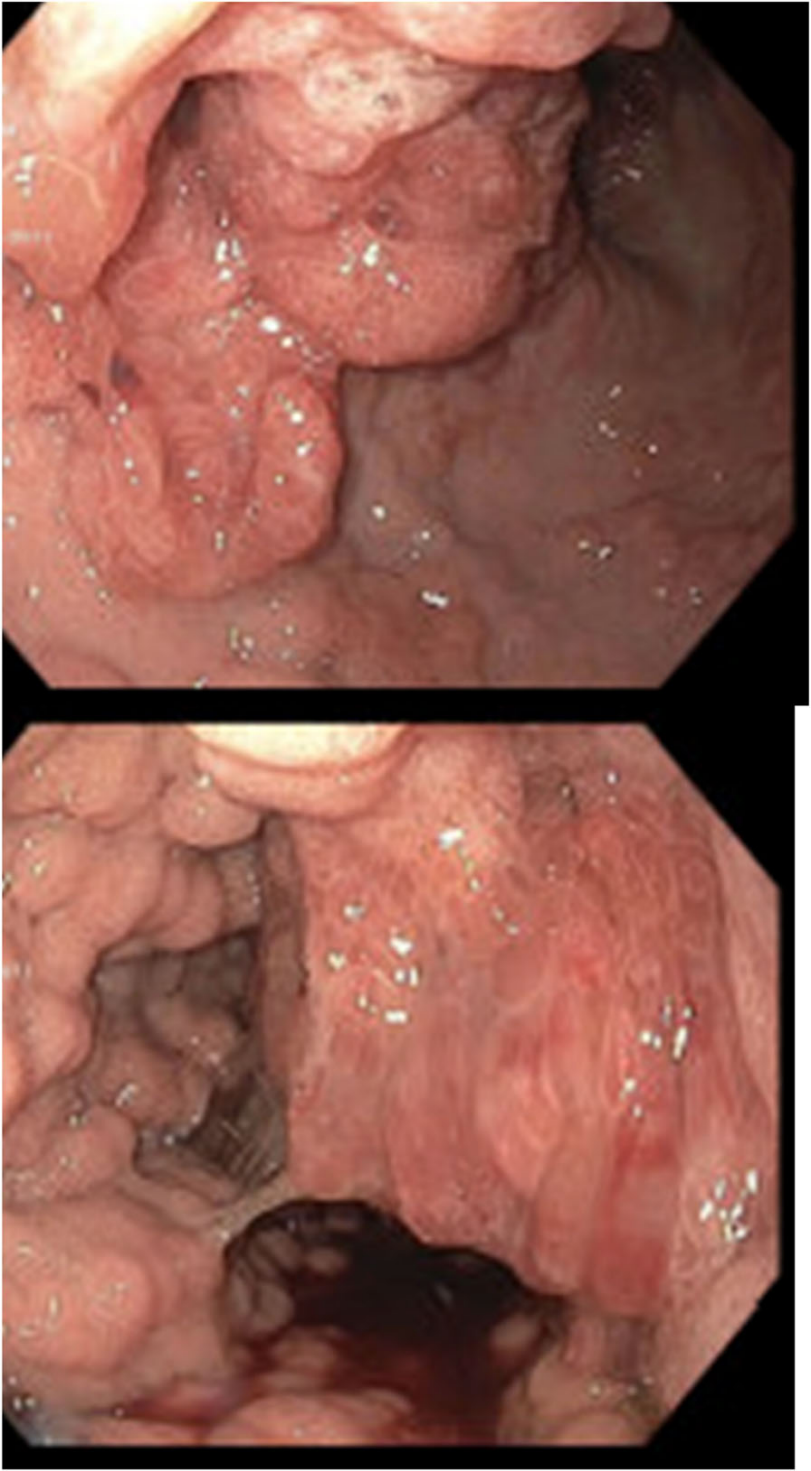
Esophagogastroduodenoscopy images showing raised, soft, markedly irregular gastric mucosa, extending from the edge of the gastric fundus to the gastric antrum

**Fig. 2:**
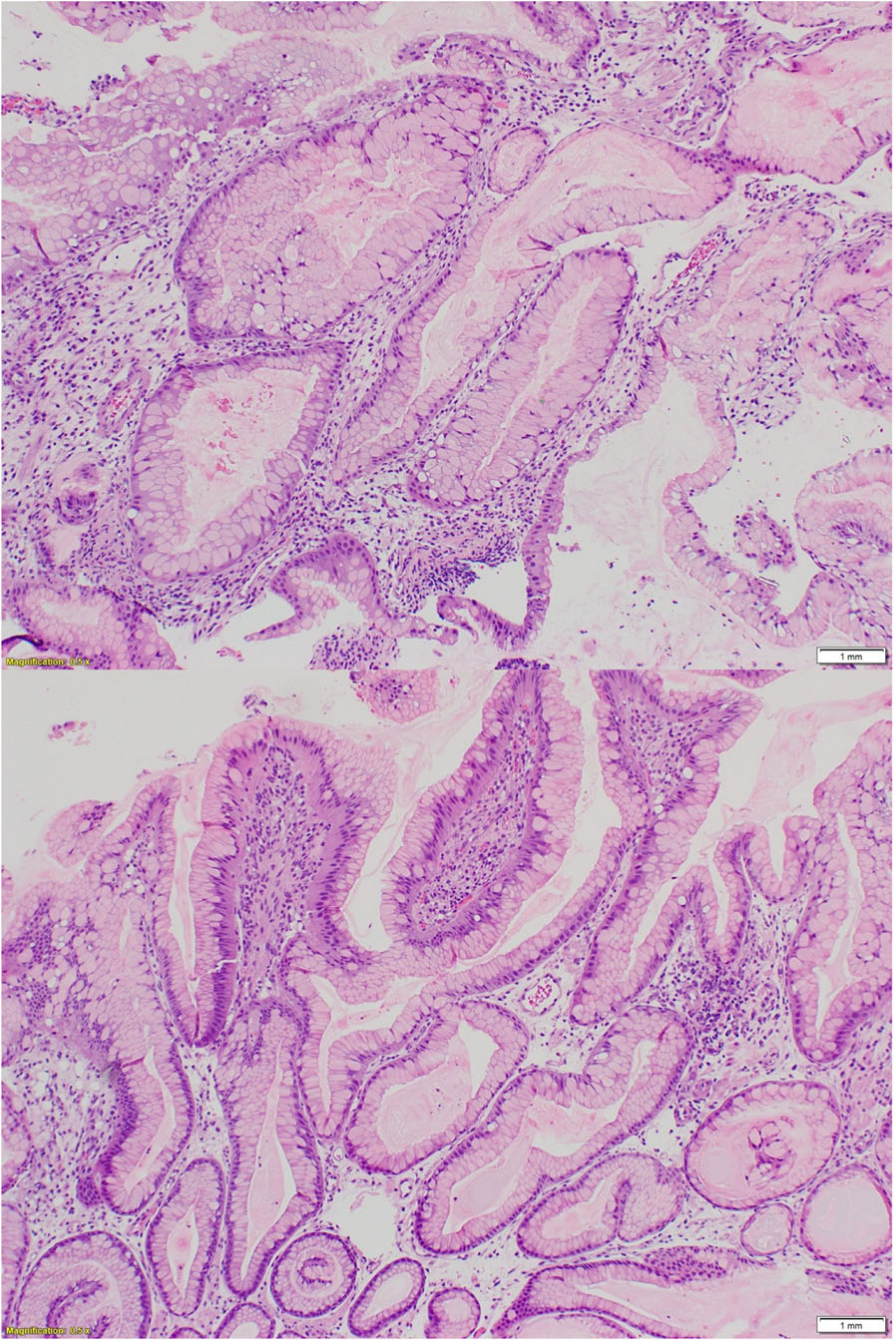
Hematoxylin and eosin-stained slide of gastric biopsy specimen. Gastric mucosa with prominent foveolar hyperplasia, focal granulation tissue, ulceration, reactive glandular changes, and evidence of chronic active inflammation. No evidence of dysplasia or malignancy

One week later, he presented again to an outside emergency department with a 3-day history of melena. An initial laboratory workup was significant for hemoglobin of 5.3 and mean corpuscular volume (MCV) of 55.7, for which he received 3 units of transfused red blood cells. Following stabilization and cessation of rivaroxaban, an abdominal computed tomography (CT) scan revealed a mass-like transmural thickening of the gastric antral and pyloric walls with tumor protrusion into the duodenal bulb. Also visualized were multifocal bilateral segmental and subsegmental pulmonary emboli, as well as a non-occlusive thrombus extending from his right renal vein into his suprarenal inferior vena cava (IVC). He underwent placement of an IVC filter. Subsequent upper endoscopy revealed diffusely irregular, raised gastric mucosa across the entire gastric body, with the appearance of a soft carpeted mass (Fig. 1). This finding was suggestive of malignancy. A biopsy specimen of the mass showed gastric mucosa with prominent foveolar hyperplasia, focal granulation tissue, ulceration, reactive glandular changes, and evidence of chronic active inflammation (Fig. 2). However, despite the suspicious gross appearance, there was no evidence of dysplasia or malignancy. Immunostaining was negative for *Helicobacter pylori*. Based on these findings, a probable diagnosis of MND was established. He was discharged on high-dose orally administered pantoprazole and scheduled for repeat upper endoscopy in 1 month.

Approximately 3 weeks later, he once again presented to an outside emergency department with the chief complaint of lumbar and suprapubic pain. An abdominal CT scan again showed a non-occlusive thrombus of his right renal vein, which was now noted to extend slightly above the IVC filter. Also visualized were abdominal varices and cavernous transformation of his portal vein suggestive of previous portal vein thrombosis. Upon this finding, he was admitted to our institution as a direct transfer for symptomatic renal vein thrombosis. A low-dose heparin drip was initiated immediately. Following transfusion of 1 unit of packed red blood cells, his hemoglobin remained between 7.2 and 7.6, and he had no ongoing melena. A comprehensive metabolic panel was notable for serum albumin of 2.4. A serum gastrin level was also elevated to 244 (upper limit of normal, 150). During his admission, he developed painful bilateral lower extremity and scrotal edema, which was managed non-pharmacologically. An urgent repeat upper endoscopy was recommended, but he declined this procedure due to concerns about the risks of endoscopic mass removal. Further intervention was deferred to his primary gastroenterologist, although treatment with cetuximab was discussed as an eventual option. He was bridged to orally administered anticoagulation without incident and was discharged on apixaban 5 mg twice daily for an indefinite duration. During a several-hour window when he was not anticoagulated, further laboratory evaluation of his hypercoagulability was performed; the results are shown in Table 1.

**Table 1:** Results of in-patient thrombophilia workup

Test	Result	Normal range	Comment
DRVVT*	41.6 seconds	28.9–36.8 seconds	Suggests presence of “lupus-like” coagulation inhibitor
STACLOT-LA** delta	14.7 seconds	0.0–10.0 seconds	Suggests presence of “lupus-like” coagulation inhibitor
Anti-cardiolipin panel	Negative	Negative	
Factor V Leiden assay	Negative	Negative	
Protein C activity	46%	70–130%	Decreased
Protein S, functional	46%	77–143%	Decreased
Antithrombin III	64%	80–120%	Decreased
Beta-2 glycoprotein IgG	< 9 units	0–20 units	
Factor V assay	97%	65–150%	
Factor VII assay	113%	60–150%	
Factor VIII assay	448%	50–180%	Increased

*Dilute Russell viper venom time

**Hexagonal (II) phase phospholipid clotting assay

At 1-month follow-up with his primary care physician, he was tolerating apixaban well, with no further gastrointestinal (GI) bleeding. He was adhering to a high-protein diet with subjective and objective improvement in his edema. His hemoglobin improved to 8.8 and his albumin to 3.2. There were no other significant changes in his laboratory results. He continued to decline repeat endoscopy or mass removal. However, he expressed plans to seek care from an out-of-state oncologist and/or gastroenterologist with expertise in treating MND.

## Discussion

There are several remarkable aspects to the above presentation. First, MND itself is quite rare, as discussed earlier. On endoscopy, the gross appearance of our patient’s gastric lesion was initially most suggestive of malignancy; his hypoalbuminemia, hypercoagulability, hypochlorhydria, and GI bleeding could all have been consistent with gastric cancer as well as MND. The pretest probability for gastric cancer was especially high given his history of juvenile polyposis syndrome, which is known to increase the risk of malignancy throughout the GI tract [21]. In this case, the diagnosis of MND was not suspected until highly suggestive histopathologic findings were observed. Second, descriptions of unprovoked venous thrombosis associated with protein-losing gastropathy are exceedingly rare. This reflects the low incidence of MND as well as the highly atypical presentation illustrated in this case. Even more unusual, our patient presented with a lower extremity DVT as the initial sign of MND; GI bleeding only began after anticoagulation was initiated. Our patient had no preceding abdominal pain, nausea, early satiety, or weight loss, at least one of which is present in most reported cases of MND.

The single most unusual facet of this case is the development of an acquired thrombophilia associated with MND. Currently, there is no literature that clearly describes the pathogenesis of acquired thrombophilia in this particular setting. However, our patient did present with several laboratory abnormalities that could certainly explain his recurrent venous thrombosis. Such laboratory abnormalities are known to contribute to hypercoagulability in other, more common, systemic diseases. In particular, derangements of hemostasis that occur in other hypoalbuminemic states, such as nephrotic syndrome and inflammatory bowel disease (IBD), provide a foundation for understanding our patient’s course.

A brief review of normal hemostatic physiology will be pertinent to the discussion that follows. Normal hemostasis occurs in two stages, with primary hemostasis mediated by platelet adhesion and aggregation, and secondary hemostasis mediated by the clotting factors numbered II–XII. Endothelial injury leads to the exposure of tissue factor, which stimulates the proteolytic activation of factor II (thrombin). This stimulus is greatly amplified by the release of various platelet-derived mediators upon platelet aggregation. Once activated thrombin is generated, it directly activates factor V, factor VII (which in turn activates factor X), and factor XI (which in turn activates factor IX). The end result of the clotting cascade is the activation of fibrinogen to fibrin, which stabilizes the platelet plugs formed in primary hemostasis. The action of this cascade is delicately counterbalanced by endogenous anticoagulant proteins such as antithrombin III, protein C, and protein S. When the zymogen forms of these proteins bind to activated clotting factors, they themselves are proteolytically activated and serve as a “check” upon secondary hemostasis. Given this physiology, it is clear that decreased anticoagulant protein activity and/or increased procoagulant protein activity (whether hereditary or acquired) may lead clinically to a hypercoagulable state. The most common hereditary thrombophilia is factor V Leiden, in which a mutated form of factor V is resistant to cleavage by protein C; others include congenital antithrombin III deficiency and prothrombin gene mutation. These were considered less likely in our patient due to the timing and severity of his presentation, and factor V Leiden was specifically ruled out by laboratory testing. Causes of acquired thrombophilia include acquired antithrombin III deficiency, which has been described in a variety of systemic diseases and is known to increase the risk of venous thromboembolism (VTE) [22, 23]. Acquired protein C deficiency [24] and protein S deficiency [25] in the setting of various conditions are also associated with increased thrombosis. Recent evidence has also demonstrated a predilection for VTE in patients with persistently elevated levels of factor VIII, which most commonly results from inflammation [26].

A systemic disease commonly associated with acquired thrombophilia is nephrotic syndrome, which is characterized by urinary protein loss exceeding 3 grams per 24 hours. Regardless of etiology, the various clinical manifestations of nephrotic syndrome result from damage to the glomerular filtration barrier. This leads to the selective loss of lower molecular size/weight proteins, most importantly albumin (~ 66.5 kDa), immunoglobulin G (~ 50 kDa), and antithrombin III (~ 58 kDa). Extensive urinary loss of protein S (~ 70 kDa) has also been described in the setting of nephrotic syndrome [27–29]. Conversely, proteins of higher molecular weights such as factor V (~ 330 kDa) and factor VIII (~ 200 kDa), are generally not lost in the urine. An immediate consequence of this selective protein loss is an imbalance in the levels of endogenous procoagulant and anticoagulant proteins. However, there is also evidence of compensatory dysregulation of hepatic protein synthesis. Although incompletely understood, this dysregulation is believed to be triggered by albumin depletion, and it results in the synthesis of fibrinogen, factor V, and factor VIII out of proportion to urinary losses. This results in a more profound pro-thrombotic state and a greatly increased incidence of VTE including, but not limited to, renal vein thrombosis [30–32]. The hypoalbuminemia and intravascular volume depletion seen in nephrotic syndrome may also lead to relative platelet [33] and red blood cell [34] hyperaggregation, further increasing thrombotic risk.

Patients with IBD also have an elevated risk of VTE, up to four times that seen in the general population [35]. Similar to patients with nephrotic syndrome, decreased serum levels of albumin, immunoglobulins, and antithrombin III have been frequently observed in patients with IBD, as well as increased levels of factor V, factor VII, and factor VIII [36–40]. Protein C deficiency has also been reported in patients with IBD and thromboembolic disease [41]. IBD represents a systemic inflammatory state, which itself is a known risk factor for elevated factor VIII and VTE. However, there is no known association of antithrombin III, factor V, or factor VII levels with active inflammation. In addition, a significantly elevated risk of VTE has been reported even during disease remission [42]. It is therefore likely that other mechanisms are also involved in the thrombophilia of IBD. In particular, many patients with IBD have a clinically significant protein-losing enteropathy due to increased lymphatic and mucosal permeability [43–45]. As in nephrotic syndrome, hypercoagulability in protein-losing enteropathy is probably driven by the loss of antithrombin III and, to a lesser extent, protein C and protein S [46–48]. This mechanism is similar to that which occurs in patients with protein-losing enteropathy secondary to Fontan-type cardiac procedures [49, 50].

Returning to our patient, we reiterate that he was found to have decreased antithrombin III, decreased protein C and S, and increased factor VIII, in addition to profound hypoalbuminemia. These results provide evidence of an imbalance between procoagulant and anticoagulant proteins, a condition which clearly explains his acquired thrombophilia. Further, given the degree of hypoalbuminemia, it is reasonable to conclude that our patient’s coagulation abnormalities developed via similar mechanisms as in nephrotic syndrome and IBD. In particular, we believe that the significant decrease in antithrombin III is a direct result of ongoing protein loss and is unlikely to be an artifact of active bleeding or systemic illness. Our patient also presented with a low level of serum protein C activity, a likely contributor to his hypercoagulability, yet protein C levels tend to be normal or even increased in nephrotic syndrome [51, 52]. This could be explained by slight differences in the selectivity of protein loss in different disease states—that is, if protein C may leak through damaged gastric mucosa more easily than a damaged glomerular filtration barrier. This is supported by the fact that protein C deficiency is observed in IBD more often than in nephrotic syndrome, as discussed above. Some degree of protein C deficiency can occur in the setting of severe inflammation, such as in patients with sepsis and multi-organ dysfunction [53]. However, given our patient’s fairly stable general appearance, the degree of his protein C deficiency suggests an additional cause besides inflammation. We did not exclude a concomitant hereditary protein C deficiency with absolute certainty, although this is highly unlikely to present with multiple thromboses of sudden onset in middle age. Our patient’s factor V level was within normal limits, although it is typically elevated in nephrotic syndrome and IBD. Because elevated factor V is a result of upregulated hepatic protein synthesis, which takes time to occur, studies obtained later in the disease course may have revealed increased factor V. However, our patient had experienced a sufficient duration of protein loss to present with a serum albumin of 2.4. It is possible that GI protein loss in MND does not affect hepatic protein synthesis in the same way as nephrotic syndrome, although this hypothesis would require further investigation. In contrast, levels of factor VIII, which is an acute-phase inflammatory reactant, could be expected to rise in a hospitalized patient even in the absence of hepatic protein over-synthesis. This rise was in fact observed in our patient.

Two important clinical questions were difficult to answer conclusively based on our patient’s course. First, the connection between this patient’s MND and his history of childhood juvenile polyposis syndrome is uncertain. Juvenile polyposis syndrome is an autosomal dominant disorder characterized by multiple hamartomatous polyps, nearly always involving the colon. However, 14% of patients with this syndrome also have gastric involvement [54]. An entity known as “massive gastric juvenile-type polyposis” has also been described in patients with and without known juvenile polyposis syndrome [55]. In addition, some reports have described an association between juvenile polyposis syndrome and MND and hypothesized that they may be manifestations of the same disease process [56, 57]. Our patient’s diagnosis of juvenile polyposis syndrome was remote, so we could not verify whether he was evaluated for gastric polyps at the time of colectomy or whether he ever received surveillance upper endoscopy. It is possible that further immunohistochemical or genetic testing would change the diagnosis from MND to juvenile or “juvenile-type” gastric polyposis. However, the resulting protein-losing gastropathy and hematologic sequelae would almost certainly be the same, regardless of diagnostic nomenclature.

Second, and more importantly for the purposes of this report, we could not easily explain the positive result for lupus-like anticoagulants. This represents another potential contributor to our patient’s hypercoagulable state, as the risk of unprovoked thrombosis in patients with antiphospholipid antibody syndrome is well characterized, with or without concomitant systemic lupus erythematosus (SLE). Antiphospholipid antibody syndrome has never been documented in association with MND, and our patient had no other manifestations of SLE. To the best of our knowledge, there are no other systemic autoimmune diseases that commonly present with protein-losing gastropathy. Elinav *et al.* described one patient whose initial presentation of SLE was the development of hyperplastic gastropathy [58]. While it would be reasonable to monitor our patient for signs of SLE in the future, this would remain a very unlikely development. False positive antiphospholipid antibody results have been described in patients who have received rivaroxaban within 24 hours and possibly up to several days [59–61]. To the best of our knowledge, however, our patient last received rivaroxaban several weeks prior to the antiphospholipid assay, so we consider this an unlikely explanation. Interestingly, the presence of low serum fibrinogen has been noted to interfere with the dilute Russell viper venom assay for lupus anticoagulant [62]. We did not measure our patient’s serum fibrinogen, but it was probably low in the presence of acute thrombosis. Ultimately, therefore, we believe that laboratory artifact was the most likely explanation for the positive lupus-like anticoagulant.

## Conclusions

In summary, we present the case of a 40-year-old man who developed acquired thrombophilia secondary to a protein-losing gastropathy. This case highlights an extremely rare cause of thrombophilia, made more notable by the fact that unprovoked venous thrombosis was the initial disease manifestation. We underscore the importance of maintaining a broad differential diagnosis when evaluating a patient with acquired hypercoagulability. Our patient’s case was also complicated by the development of GI bleeding when therapeutic anticoagulation was initiated. His simultaneous bleeding and hypercoagulability presented a distinct clinical challenge, which ultimately necessitated in-patient observation and unfractionated heparin therapy.

The strengths of this case report include its novelty; we describe a rare disease process involving the GI system, with even more unusual hematologic manifestations. The workup of our patient was quite thorough, as it involved multiple hospital admissions and subspecialty evaluations. In addition, coagulation studies were drawn after all anticoagulants had been held for a sufficient duration, minimizing the likelihood of medication-induced abnormalities. The limitations of this case report include sub-optimal follow-up, largely because our patient refused repeat endoscopy and sought further care out-of-state. There are also several causes of thrombophilia that were not assessed in this patient—for example, increased activity of prothrombin or homocysteine. At the discretion of the treating clinicians, these tests were withheld because they were felt to be low yield and unlikely to influence management.
